# Comprehensive analysis of immunogenic cell death associated genes expression, tumor microenvironment, and prognosis in hepatocellular carcinoma

**DOI:** 10.3389/fphar.2023.1122011

**Published:** 2023-03-14

**Authors:** Jiankang Xiang, Chuan Liu, Qingmin He, Pengzhan He, Weiguo Dong

**Affiliations:** ^1^ Department of Gastroenterology, Renmin Hospital of Wuhan University, Wuhan, China; ^2^ Henan Key Laboratory of Helicobacter Pylori and Microbiota and Gastrointestinal Cancer, Marshall Medical Research Center, the Fifth Affiliated Hospital of Zhengzhou University, Zhengzhou, China

**Keywords:** hepatocellular carcinoma, immunogenic cell death, prognosis, tumor microenvironment, bioinformatics analysis

## Abstract

**Background:** Immunogenic cell death (ICD) plays an important role in the development of cancers. This study attempted to explore the role of ICD in the prognosis of hepatocellular carcinoma (HCC).

**Methods:** Gene expression and clinical data were downloaded from The Cancer Genome Alas and Gene Expression Omnibus dataset. The immune/stromal/Estimate scores of the tumor microenvironment (TME) were calculated by ESTIMATE and CIBERSORT algorithms. Kaplan-Meier analysis, functional enrichment analysis, least absolute shrinkage and selection operator (LASSO) analysis, and univariate and multivariate Cox regression analysis were used for prognostic gene screening and prognostic model construction. The correlation of immune cell infiltration and risk scores was analyzed as well. Molecular docking was used to explore the relevance of related genes to anti-cancer drugs.

**Results:** Ten ICD associated differentially expressed genes in HCC were found, and all of them had good predictive ability for HCC. ICD gene high amount of expression group was associated with poor prognosis (*p* = 0.015). The TME, immune cell infiltration and gene expression were different between ICD high and low groups (all *p* < 0.05). Six ICD associated genes (BAX, CASP8, IFNB1, LY96, NT5E and PIK3CA) which could predict the survival status were identified and used to construct the prognostic model for HCC. A risk score was calculated and it could be used as an independent prognostic factor in HCC patients (*p* < 0.001). In addition, the risk score had a positive correlation with macrophage M0 (r = 0.33, *p* = 0.0086). Molecular docking indicated that sorafenib could bind strongly to the target protein, representing that sorafenib may exert anticancer effects through these six ICD associated genes.

**Conclusion:** This study established a prognostic model including six ICD associated genes for HCC, which may deepen our understanding of ICD and guide therapy for HCC patients.

## Introduction

Hepatocellular carcinoma (HCC) ranks sixth in the world among the common cancers (Konyn et al., 2021), and the most common type of liver cancer is HCC. Hepatis B or C virus infection and alcohol abuse are the most common causes of HCC (Villanueva, 2019). Liver resection or transplantation, locoregional therapies such as radiofrequency ablation, trans-arterial therapies like chemoembolization and external beam radiation therapy are main treatment measures for liver cancer (Ren et al., 2020). Tumor-specific T cell lymphocytes and other immune cells can infiltrate HCC because it is immunogenic, so immunotherapy may play an important role in HCC by inducing a tumor-specific immune response (Lawal et al., 2021).

Immunogenic cell death (ICD) is a functionally unique form of stress-driven regulated cell death (RCD), by which cytotoxic T lymphocyte and inflammatory response are activated and adaptive immune response and immunological memory, as well as antigenicity and adjuvanticity, are initiated (Galluzzi et al., 2019). Exposure and release of damage-associated molecular patterns (DAMPs) consist of the immunogenic characteristics of ICD. ICD associated DAMPs include released high-mobility group box 1 (HMGB1), surface-exposed calreticulin (CARL), secreted ATP and annexin A1 (ANXA1). ICD plays an important role in the development of cancers. Several studies have been conducted to explore some of the genes that influence the prognosis of HCC (Rao et al., 2021; Chen et al., 2022). However, the role of ICD associated genes in the prognosis of HCC had not been explored.

In this study, we explored the relationship between ICD and prognosis in HCC patients from a public database. Patients were divided into 2 groups according to the gene expressions of ICD, and differentially expressed genes (DEGs) and survival analysis were analyzed. In addition, immune scores, TME and risk score were also calculated. We constructed a prognostic model which could predict the prognosis and survival status in HCC.

## Materials and methods

### Data download and process

The TCGA GDC database (https://portal.gdc.cancer.gov/) is an open-access platform for various kinds of cancers. The transcriptome profiling, simple nucleotide variation and other clinical data can be used in TCGA database. We set the primary site as liver, and choose the program for TCGA and the project for TCGA-LIHC. Metadata and cart profiles were retrieved, including 447 patients (374 tumor samples and 50 normal samples). Clinical data were downloaded and processed, which contained the age, gender, survival time and survival status, grade and stages of patients. Simple nucleotide mutation variation and masked somatic mutation were acquired from TCGA, from which we could find the start position and end position of mutation gene, chromosome, variant classification and variant type. Then we calculated the tumor mutation burden and mutation quantification. ICD associated gene expressions were obtained. The validation cohort (GSE 10186) was obtained from GEO datasets (https://www.ncbi.nlm.nih.gov/gds/?term=), which included 118 resected HCC specimens and their corresponding clinical data. The probes were labeled with gene symbols based on the annotation information on the platform.

### Enrichment analysis and protein-protein interactions (PPIs) network of DEGs

We set the criteria of |log2-fold change (FC)| > 1 and adjusted *p* < 0.05 as upregulated and downregulated DEGs. To understand the biological functions and pathways of ICD associated genes, Gene Ontology (GO) functional enrichment and Kyoto Encyclopedia of Gene and Genomes (KEGG) pathway analyses were performed using the ClusterProfiler package in Bioconductor (https://www.bioconductor.org/packages/release/bioc/html/clusterProfiler.html). To explore the underlying molecular mechanisms for the involvement of the key genes, gene set enrichment analysis (GSEA) was drawn to investigate the differences in the KEGG pathways between the high- and low-expression groups of ICD associated genes. The DEGs were imported into the Search Tool for the Retrieval of Interacting Genes/Proteins (STRING) online database (https://string-db.org/) to construct a PPI network, and we set the minimum required interaction score with medium confidence (0.400).

### Survival analysis and prognostic model construction

Kaplan-Meier method was used to construct a survival curve. The least absolute shrinkage and selection operator (LASSO) Cox regression model was used to identify and construct a prognostic model based on all genes in ICD associated prognosis genes. After enrolling each gene expression value, we calculated the formula for the risk score of each sample. The risk score formula was established by weighting the estimated regression coefficients in the LASSO analysis. In addition, the DEGs were screened to construct the nomogram prediction model. The receiver operating characteristic (ROC) curve was drawn, and the prediction ability of the model was evaluated by the area under the ROC curve (AUC).

### Tumor microenvironment and immune infiltration analysis

ESTIMATE is an algorithm that uses gene expression signatures to infer the fraction of stromal and immune cells in tumor samples. Immune score, stromal score and ESTIMATE score and their relative proportions were calculated by using ESTIMATE algorithms. “Limma” and “estimate” packages were used for this process. The Spearman correlation analysis was performed on the immune cell infiltration levels and gene expression levels. In addition, all immunohistochemistry (IHC) images were obtained from The Human Protein Atlas (HPA) online database (https://www.proteinatlas.org/).

### Molecular docking

Sorafenib, the first-line targeted drug for hepatocellular carcinoma. It is a dual-channel multi-target inhibitor. On the one hand, it inhibits VEGFR and PDGFR to block tumor angiogenesis, and on the other hand, it blocks Raf/MEK/ERK signaling pathway to inhibit tumor cell proliferation. To investigate the association of six ICD-related genes with anticancer drugs, this study searched the details of Sorafenib using CAS: 284461–73-0 and downloaded its 3D structure in the PubChem database (https://pubchem.ncbi.nlm.nih.gov/). The protein structures of six ICD-related genes were downloaded from the RCSB PDB database (https://www.rcsb.org/), and the information of the proteins was shown in Table 1. Protein was dehydrated and de-liganded in PyMOL. Sorafenib was converted to mol2 format in OpenBabel. Molecular docking was performed and the binding energy was collected in AutoDockTools 1.5.7. Finally, the docking results were visualized by PyMOL.

**TABLE 1:** Protein information of six ICD-related genes (ICD: immunogenic cell death).

Targets	PDB ID	Method	Resolution(Å)	R-Value free	R-Value work	R-Value observed
BAX	2IMS	X-RAY DIFFRACTION	1.48	0.204	0.179	0.18
CASP8	3KJQ	X-RAY DIFFRACTION	1.80	0.207	0.182	0.185
IFNB1	1AU1	X-RAY DIFFRACTION	2.20	0.283	0.223	0.223
LY96	2E56	X-RAY DIFFRACTION	2.00	0.249	0.194	0.196
NT5E	7QGO	X-RAY DIFFRACTION	2.21	0.292	0.239	0.242
PIK3CA	8EXL	X-RAY DIFFRACTION	1.99	0.225	0.187	0.188

### Statistical analysis

Statistical analysis was performed using R software (version 4.2.1). Univariate and multivariate Cox regression analysis were used to select risk-related factors and genes. Hazard ratios (HRs) and their 95% confidence intervals (CIs) were calculated by using multivariate Cox regression analysis. All statistical tests were two-sided and *p*-values of <0.05 were considered statistically significant differences.

## Results

### ICD associated DEGs

#### Identification of ICD associated DEGs

**FIGURE 1:**
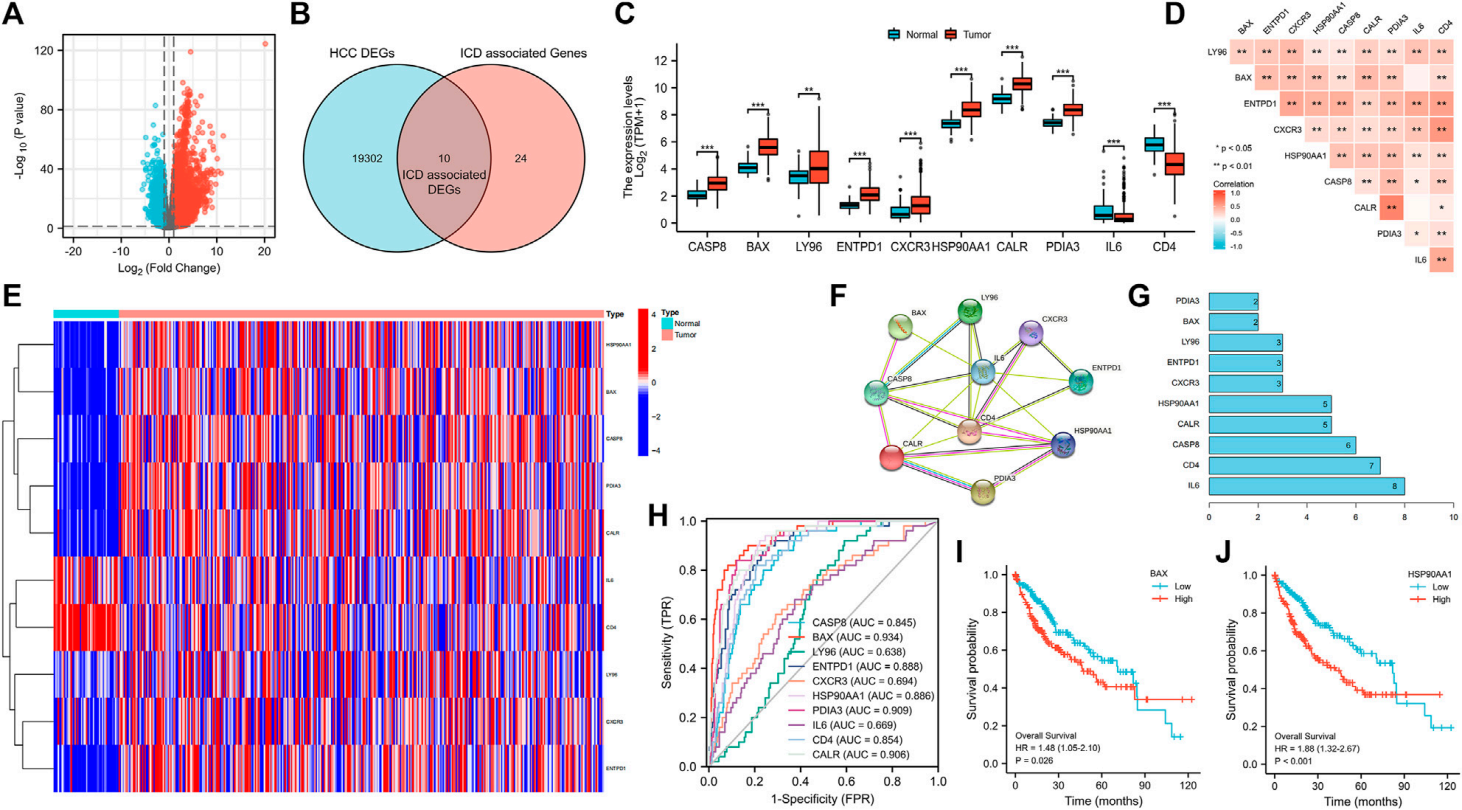
Volcano plot of HCC DEGs **(A)**, and Venn diagram **(B)**, bar chart **(C)**, correlation heatmap **(D)**, genes expression heatmap **(E)**, the protein-protein interaction networks **(F)**, genes sorted by quantity of nodes **(G)** and ROC curve **(H)** of ICD associated DEGs, and survival curves of BAX **(I)** and HSP90AA1 **(J)**. (HCC: Hepatocellular carcinoma, DEGs: Differentially expressed genes, ICD: Immunogenic cell death, ROC curve: The receiver operating characteristic curve; *p < 0.05, **p < 0.01, ***p < 0.001).

There were 19312 DEGs between HCC and normal tissues (Figure 1A), and there were 34 ICD associated genes. A total of 10 ICD associated DEGs were found (Figure 1B), among which the upregulated genes were LY96, BAX, ENTPD1, CXCR3, HSP90AA1, CASP8, CALR and PDIA3, and the downregulated genes were IL6 and CD4 (Figure 1C). The 10 DGEs were highly correlated with each other (Figure 1D), and the DEGs were shown in the clustering heatmap (Figure 1E). In addition, these 10 ICD associated DEGs had a good predictive ability for HCC (Figure 1H).

#### PPI network construction of ICD associated DEGs

From the PPI network (Figure 1F), the number of nodes was 10, the number of edges was 22, average node degrees were 4.4, and average local clustering coefficients were 0.795, with PPI enrichment *p*-value < 8.6e^−07^. DEGs sorted by quantity of nodes were drawn (Figure 1G).

#### IHC and immune infiltration analysis

**FIGURE 2:**
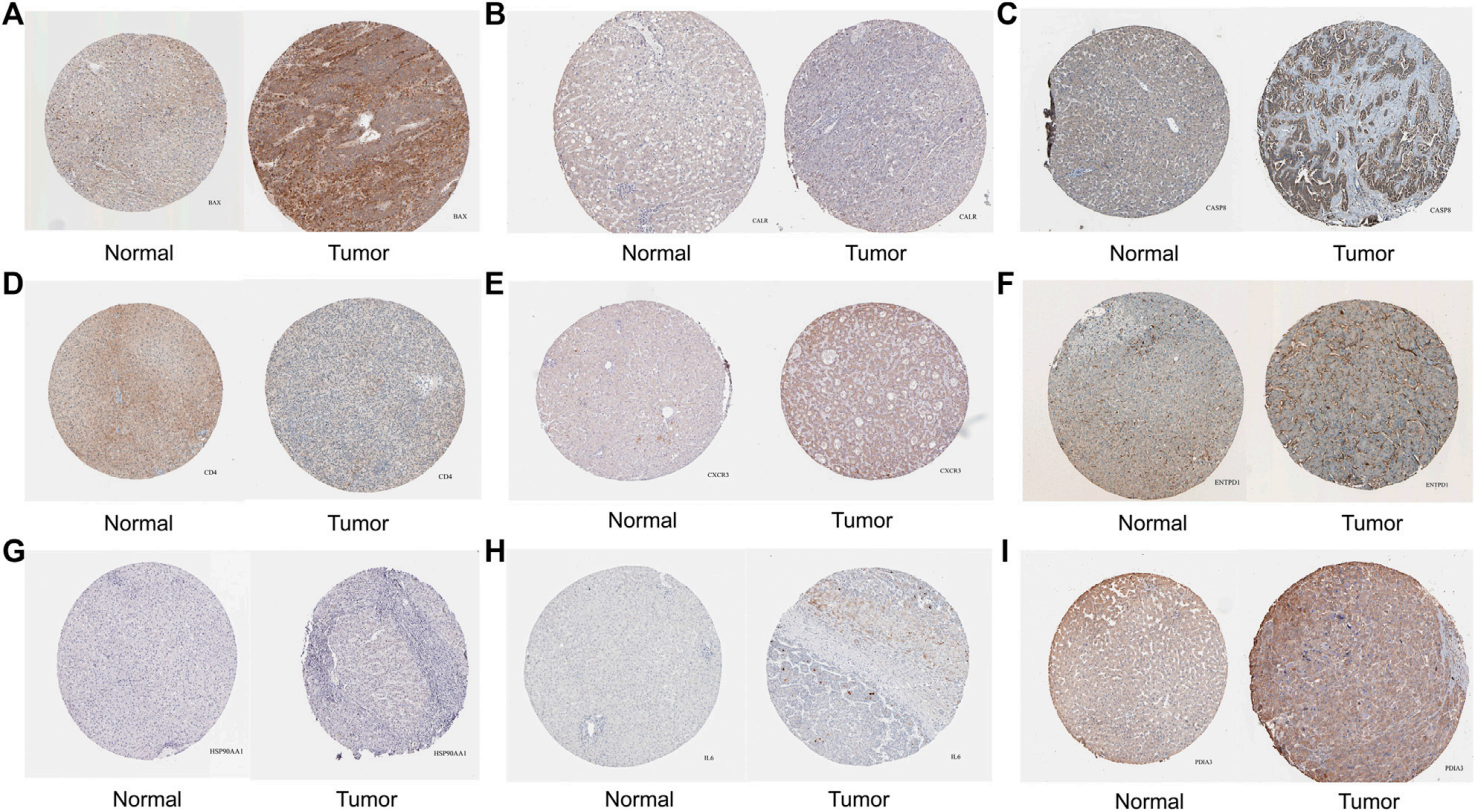
The protein level of ICD associated DEGs in normal liver and tumor tissues were detected by immunohistochemistry from the Human Protein Atlas database. [**(A)** BAX, **(B)** CALR, **(C)** CASP8, **(D)** CD4, **(E)** CXCR3, **(F)** ENTPD1, **(G)** HSP90AA1, **(H)** IL6, **(I)** PDIA3; ICD: Immunogenic cell death, DEGs: Differentially expressed genes].

From HPA, we obtained pathological pictures of ICD associated DEGs in normal liver tissues and tumor tissues in HCC (Figure 2). Moreover, all ICD associated DEGs were significantly associated with a large number of immune cells (Supplementary Figure S1).

#### Survival analysis and clinical correlation analysis

The high expression of BAX and HSP90AA1 significantly reduced the survival of HCC patients (Figures 1I, J). In addition, we found no significant correlation between these 10 ICD associated DEGs and relevant clinical characteristics (Supplementary Figure S2).

#### Construction of nomogram prediction model

**FIGURE 3:**
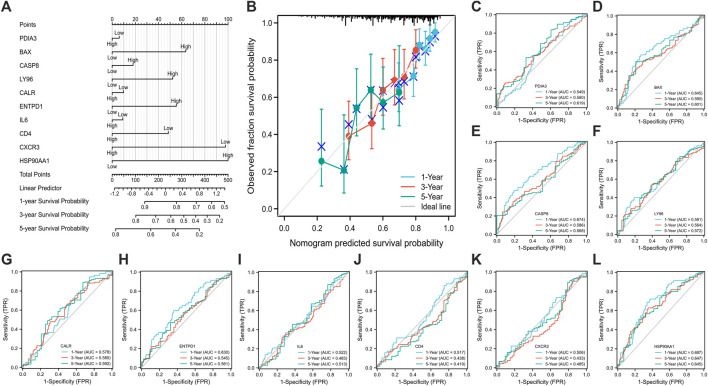
Establishment **(A)** and evaluation **(B–L)** of nomogram model. [calibration curve **(B)**, the ROC curve **(C–L)**; **(C)** PDIA3, **(D)** BAX, **(E)** CASP8, **(F)** LY96, **(G)** CALR, **(H)** ENTPD1, **(I)** IL6, **(J)** CD4, **(K)** CXCR3, **(L)** HSP90AA1; ROC: receiver operating characteristic].

The nomogram model with 10 ICD associated DEGs was constructed to predict the 1-year, 3-year, and 5-year survival of HCC patients (Figure 3A). The calibration diagram of the model showed that the calibration curve fitted well with the ideal curve (Figure 3B), indicating that the accuracy of the model was high. The ROC curve was used to analyze the predictive ability of the nomogram prediction model, and the AUC values of 10 ICD associated DEGs indicated that the predicted values were acceptable (Figures 3C–L).

### Analysis of DEGs between ICD high and low expression groups

#### ICD gene clustering and survival analysis

**FIGURE 4:**
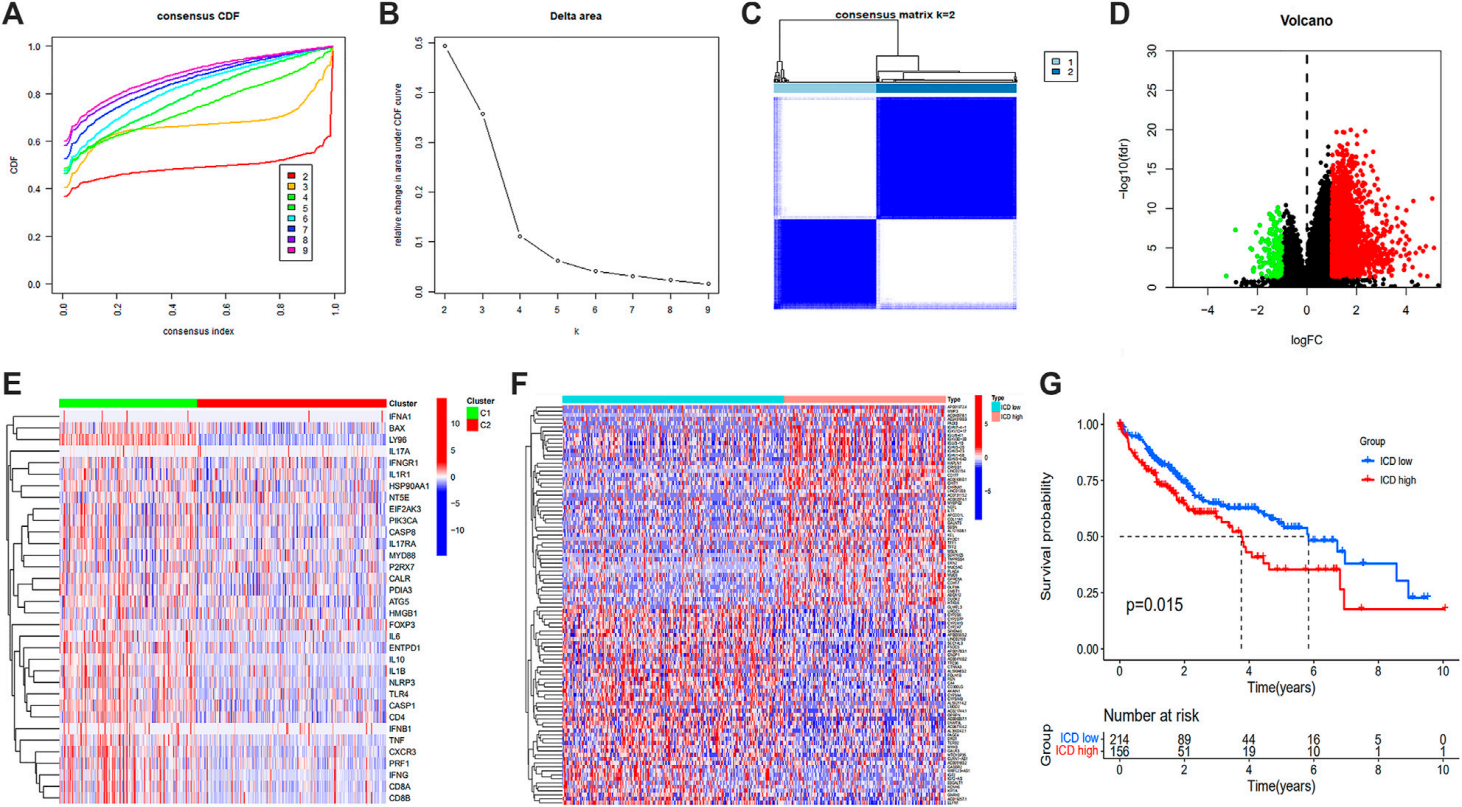
Unsupervised consensus clustering of HCC samples based on ICD associated DEGs **(A,B)**, consensus CDF curve and area under CDF curve when k = 2–9 **(C)**, two clusters according to the best consensus matrix (k = 2) **(E)**, volcano plot **(D)**, heatmap **(F)**, and Kaplan-Meier survival plot **(G)** of DEGs between ICD high and low expression groups. (HCC: Hepatocellular carcinoma, ICD: Immunogenic cell death, DEGs: Differentially expressed genes).

Samples were clustered according to ICD gene expression. We set the number of clusters = 2 to get the clustering result (Figure 4E). According to the gene expression level, the ICD gene expression level was divided into ICD high and low expression groups (Figures 4A–C). The volcano map (Figure 4D) and heat map (Figure 4F) were drawn. From the survival curve, we found that there was a statistically significant difference in survival status between ICD high and low expression groups (*p* = 0.015) (Figure 4G).

#### GO and KEGG analysis of DEGs between ICD high and low expression groups

**FIGURE 5:**
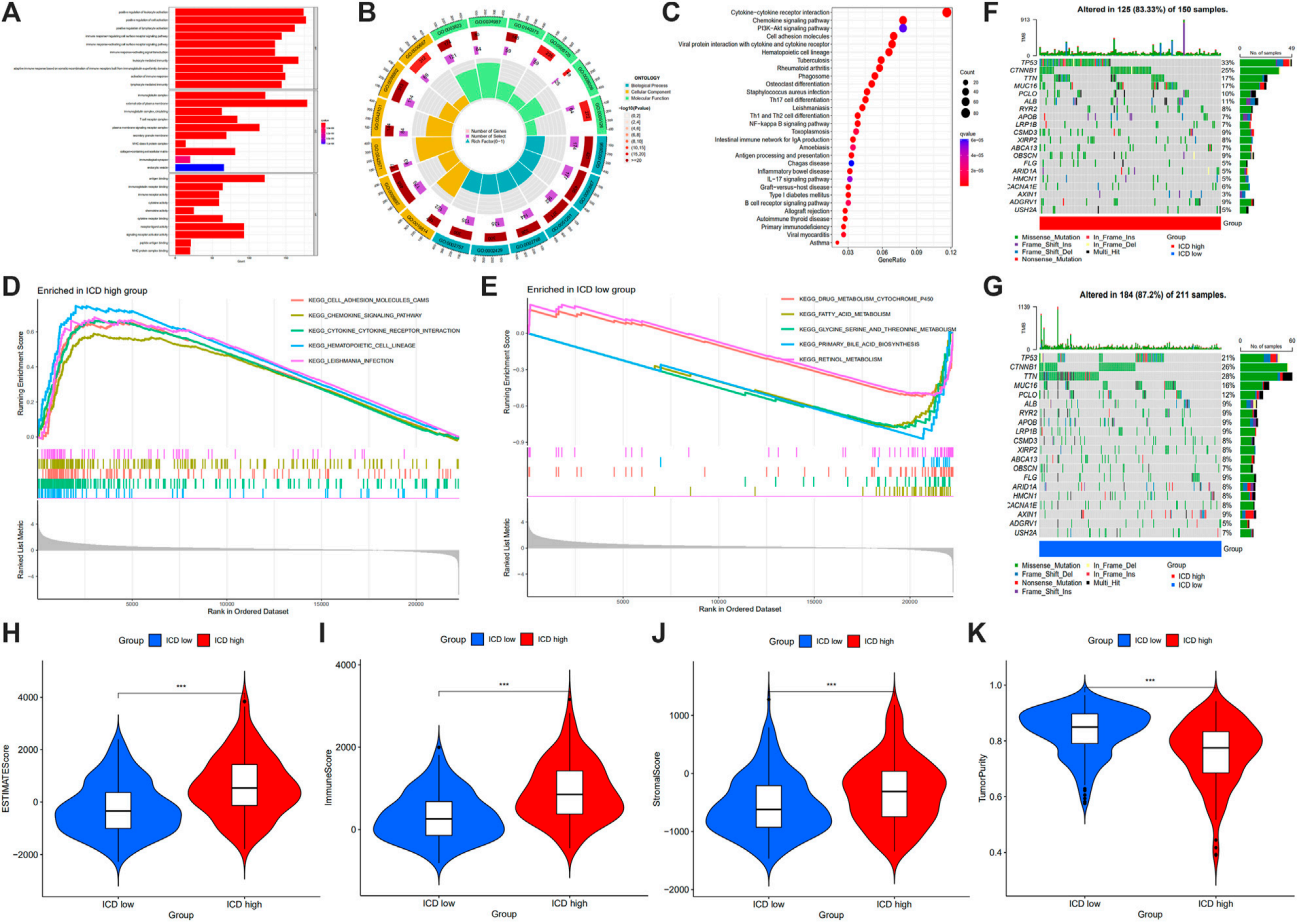
GO term **(A,B)** and KEGG pathway **(C)** analysis of DEGs between ICD high and low expression groups, and GSEA analysis **(D,E)**, maftools **(F,G)**, ESTIMATE score **(H)**, immune score **(I)**, stromal score **(J)** and tumor purity **(K)** in ICD high and ICD low group. (GO: Gene Ontology, KEGG: Kyoto Encyclopedia of Gene and Genomes, GSEA: gene set enrichment analysis, DEGs: differentially expressed genes, ICD: immunogenic cell death; *p < 0.05; **p < 0.01: ***p < 0.001).

The enrich GO function was used to enrich from biological process (BP), cellular component (CC) and molecular function (MF), respectively (Figures 5A, B). From BP, the top 3 GO terms were identified and mainly included positive regulation of leukocyte activation, positive regulation of cell activation, and positive regulation of lymphocyte activation. From CC, the top 3 GO terms included immunoglobulin complex, external side of the plasma membrane, and circulating immunoglobulin complex. From MF, the top 3 GO terms included antigen binding, immunoglobulin receptor binding, and immune receptor activity. The enrich KEGG function showed that the differential genes were enriched in cytokine-cytokine receptor interaction, chemokine signaling pathway, PI3K-Akt signaling pathway, etc (Figure 5C).

#### GSEA, maftools and gene mutation analysis

From GSEA, we could see cell adhesion molecules, chemokine signaling pathway, cytokine-cytokine receptor interaction, hematopoietic cell lineage and leishmania infection were enriched in ICD high group (Figure 5D), and drug metabolism cytochrome P450, fatty acid metabolism, glycine, serine and threonine metabolism, primary bile acid biosynthesis and retinol metabolism were enriched in ICD low group (Figure 5E). Gene mutation data were divided based on ICD high expression and ICD low amount of gene mutation, from which chromosome, base mutation and tumor samples were shown (Figures 5F, G). In the ICD gene high amount of mutation, 125 of 150 samples (83.33%) were altered, and 184 of 211 samples (87.2%) in the ICD gene low amount of mutation were altered. TP53 altered in 33% of samples in the ICD high group, whereas 21% in ICD low group. CTNNB1 altered in 25% of samples in ICD high group, and 26% in the ICD low group. TTN altered in 17% of samples in the ICD high group, and 28% in ICD low group.

#### Tumor microenvironment scores analysis

**FIGURE 6:**
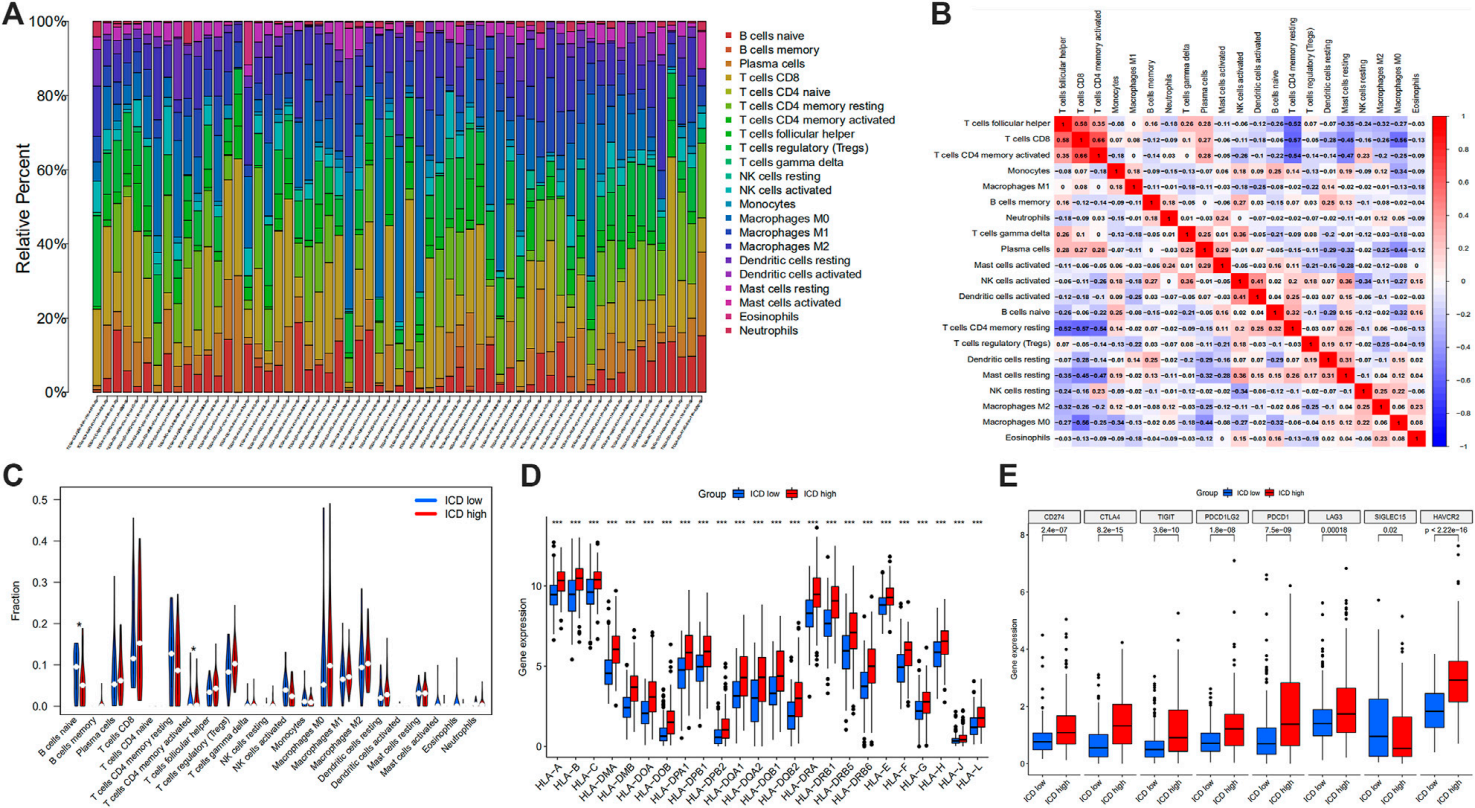
Histogram showing 22 types of TIILs in each case **(A)**, the correlations between the TIILs **(B)**, comparison of the immune cell fractions **(C)**, HLA **(D)** and immune checkpoint associated gene expression **(E)** between ICD high and ICD low groups. (TIILs: tumor-infiltrating immune cells, HLA: Human Leukocyte Antigens, ICD: immunogenic cell death; *p < 0.05; **p < 0.01: ***p < 0.001).

In the tumor microenvironment score, we found that the group with ICD high gene expressions had a higher estimate score (Figure 5H), immune score (Figure 5I) and stromal score (Figure 5J), but lower tumor purity (Figure 5K). Histograms showed 22 types of TIILs in each case (Figure 6A), and heatmaps showed correlations between TIILs (Figure 6B). From the immune cell differential analysis, we found that B cell naive and T cell CD4 memory activated were significantly different between the two groups (*p* < 0.05) (Figure 6C).

#### Human Leukocyte Antigens (HLA) and immune checkpoint gene differential analysis

HLA analysis showed that HLA-A, HLA-B, HLA-C, HLA-DMA, HLA-DMB, HLA-DOA, HLA-DOB, HLA-DPA1, HLA-DPB1, HLA-DBP2, HLA-DQA1, HLA-DQA2, HLA-DQB1, HLA-DQB2, HLA-DRA, HLA-DRB1, HLA-DRB5, HLA-DRB6, HLA-E, HLA-F, HLA-G, HLA-H, HLA-J and HLA-L in ICD gene high expression group were higher than those of ICD gene low expression group (Figure 6D). According to immune checkpoint gene differential analysis, we found that CD274, CTLA4, TIGIT, PDCD1LG2, PDCD1, LAG3 and HAVCR2 in ICD gene high expression group were higher than those of the ICD low expression group whereas SIGLEC15 was lower in ICD high group (Figure 6E).

### ICD associated prognosis genes

#### Find ICD associated prognosis genes

**FIGURE 7:**
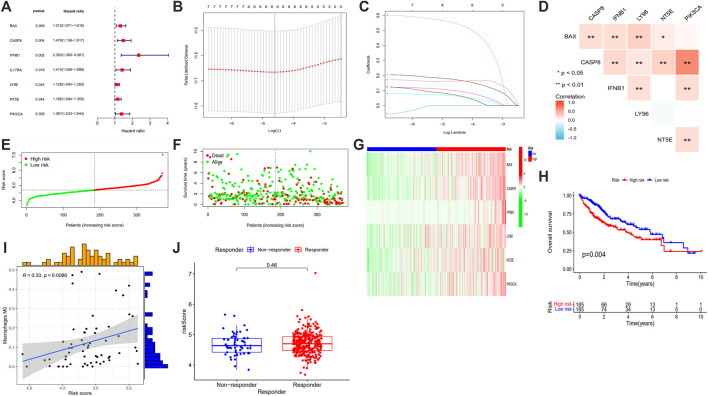
Forest plots of univariate prognostic analysis **(A)**, LASSO regression analysis **(B)**, coefficient profile plot **(C)**, correlation heatmap **(D)** of 6 ICD associated prognosis genes, risk score plot **(E)**, survival status scatter plot **(F)**, heatmap **(G)** and survival curve **(H)** of high risk and low risk groups, correlation between the risk score and Macrophage M0 **(I)** and risk score in responder and non-responder groups in HCC who had received immune-checkpoint inhibitors **(J)**. (LASSO: least absolute shrinkage and selection operator, ICD: immunogenic cell death, HCC: hepatocellular carcinoma; *p < 0.05; **p < 0.01: ***p < 0.001).

All data in the GEO database were transformed. The same gene in GEO and TCGA were intersected and rectified, and ICD associated gene expressions were output. From the forest map based on univariate analysis, we found that BAX, CASP8, IFNB1, IL17RA, LY96, NT5E and PIK3CA were the 7 risk genes (all *p* < 0.05) (Figure 7A). LASSO regression model of HCC patients showed that the best number of ICD associated prognosis genes in the model was 6, which were BAX, CASP8, IFNB1, LY96, NT5E and PIK3CA, and the formula was risk scores = gene expression of BAX*0.1738 + gene expression of CASP8*0.1628 + gene expression of IFNB1*0.5482 + gene expression of LY96*0.0646 + gene expression of NT5E*0.1006 + gene expression of PIK3CA*0.0723 (Figures 7B, C). There was a good correlation between these 6 ICD associated prognosis genes in HCC (Figure 7D).

#### Risk curve and survival analysis

The samples were sequenced according to risk scores, the lower risk group was labeled blue and the higher risk group was labeled red color (Figure 7G). When making the survival status map, patients alive were dotted with green and patients dead were dotted with red points (Figure 7F). From the risk curve and survival status map, we found that with the risk scores increased, patients’ overall survival time decreased (Figures 7E, F). From the survival curve, the overall survival of patients in higher risk group was shorter than those of lower risk group (Figure 7H).

#### Correlation analysis of clinical characteristics

**FIGURE 8:**
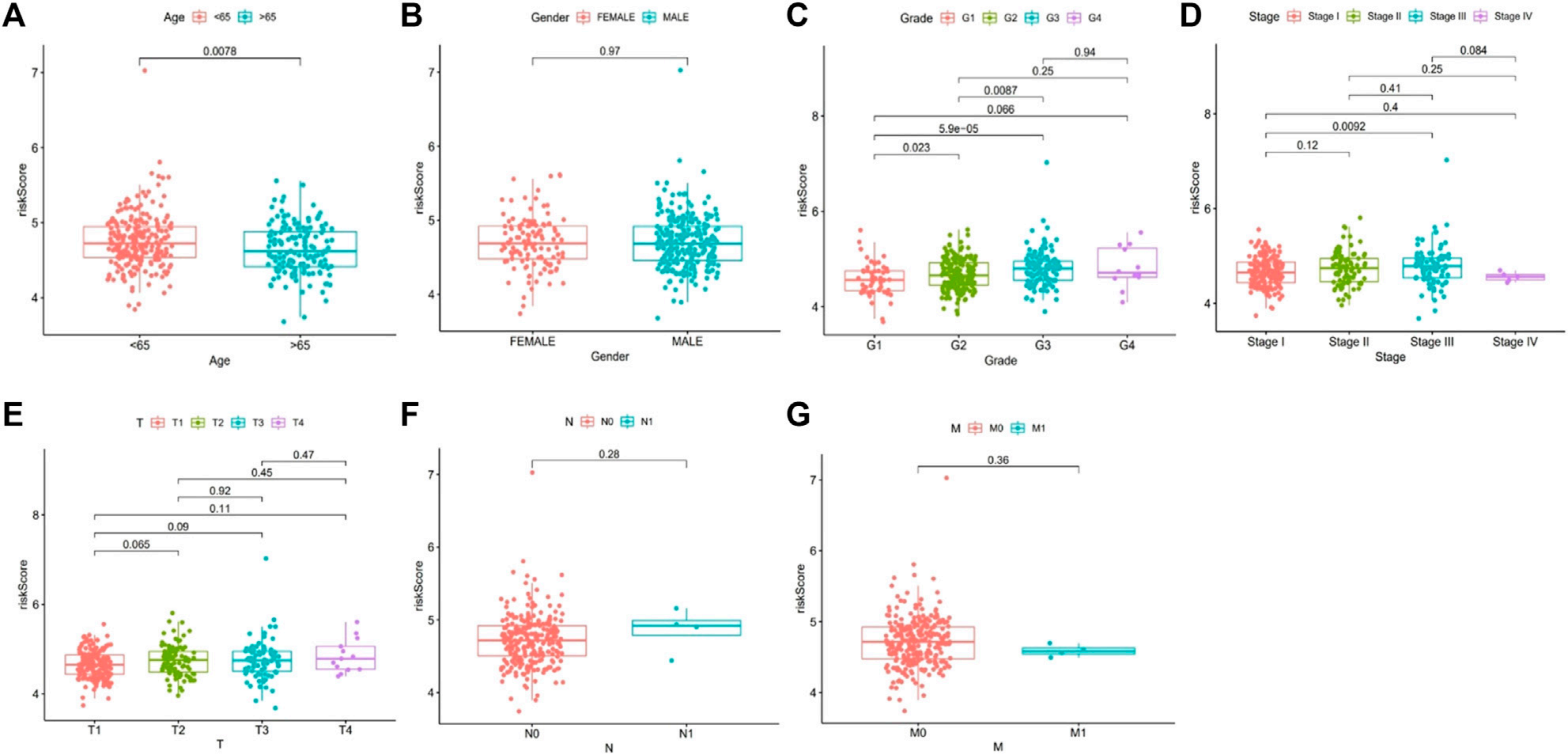
Correlation analysis of risk score and related clinical characteristics. [**(A)**: age, **(B)** gender, **(C)** grade, **(D)** stage, **(E)** T stage, **(F)** N stage, **(G)** M stage].

The risk score was correlated with age (> 65 years old vs. < 65 years old), grade (G2 vs. G1; G3 vs. G1; G3 vs. G2), and stage (III vs. I) (all *p* < 0.05) (Figure 8).

#### Correlation analysis of immune cells

We obtained the correlation between risk scores and immune cells, and scatter plots and spearman correlation values were constructed for immune cells with *p* < 0.05. It showed that macrophage M0 was positively associated with risk score (r = 0.33, *p* = 0.0086) (Figure 7I). To explore the role of the risk score in differentiating responders and non-responder in HCC who had received immune-checkpoint inhibitors, we obtained TIDE data from http://tide.dfci.harvard.edu and we found that the risk score was not significantly different (Figure 7J).

#### Independent prognosis analysis

**FIGURE 9:**
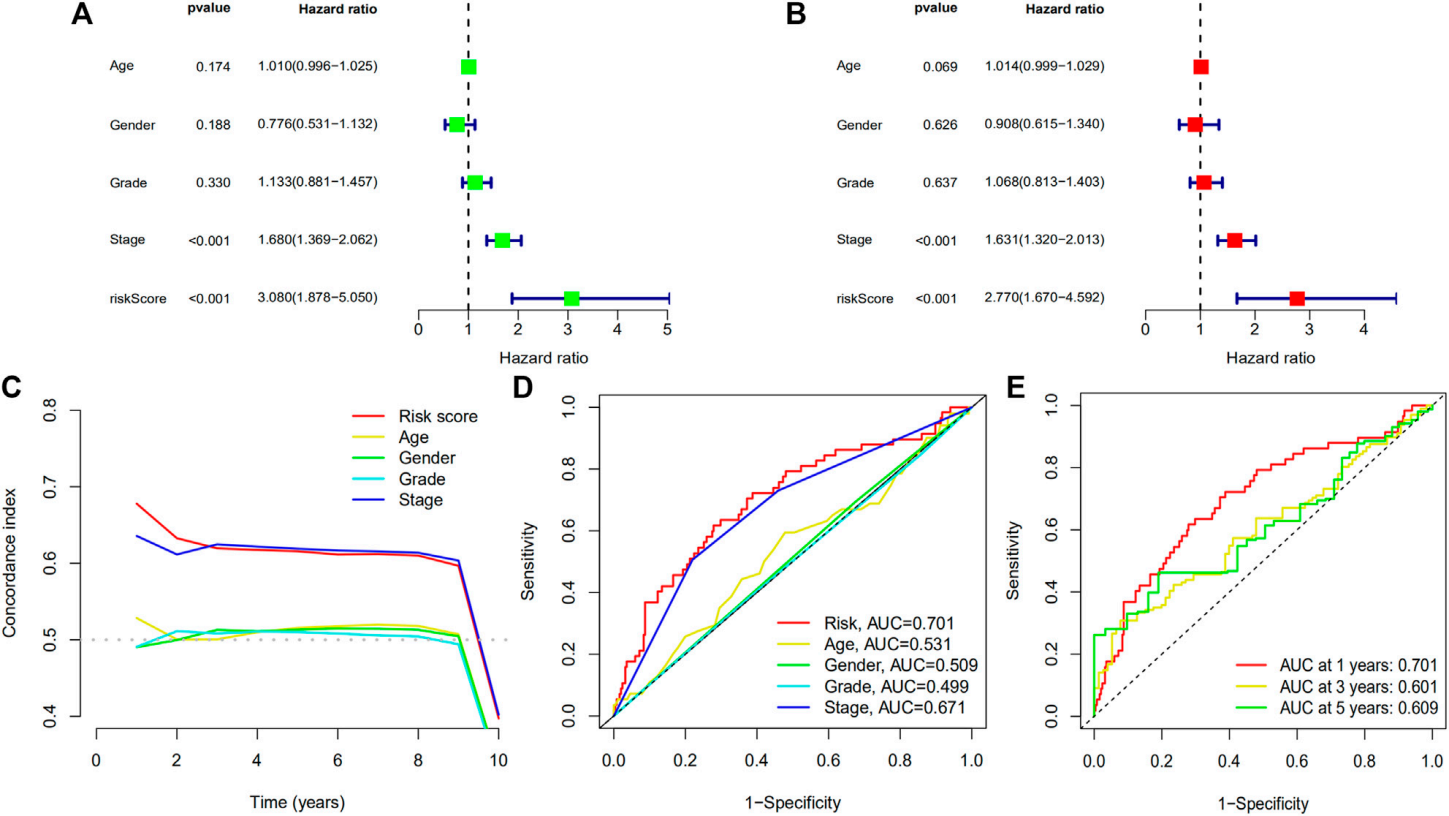
Forest plots of univariate prognostic analysis for risk score **(A)** and multivariate prognostic analysis **(B)**, and concordance index **(C)** and ROC curve **(D)** of different variables, and ROC curve **(E)** of prognostic model. (ROC: receiver operating characteristic).

Based on univariate prognosis analysis, it showed that stage and risk score were independent risk factors for prognosis (all *p* < 0.001) (Figure 9A). From multivariate prognosis analysis, we found stage and risk score were independent risk factors for prognosis (all *p* < 0.001) (Figure 9B). Concordance index and AUC showed that risk score and stage had a significantly higher predictive performance for prognosis (Figures 9C, D), and this model had an acceptable performance for predicting the prognosis of HCC patients in 1 year, 3 years, and 5 years (Figure 9E).

#### Molecular docking validation of sorafenib and protein

**FIGURE 10:**
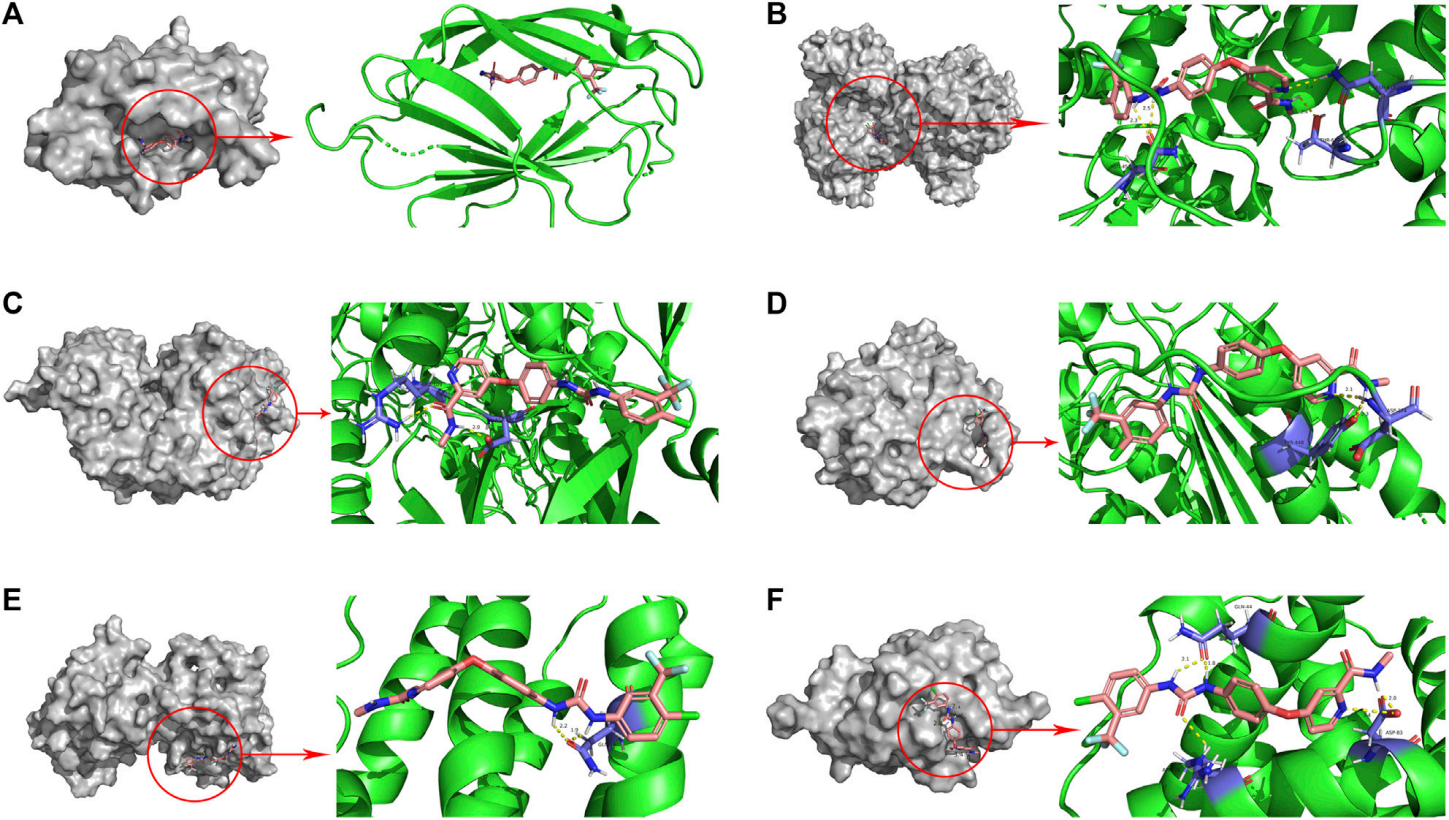
Molecular docking pattern of sorafenib and core target protein. [**(A)** sorafenib-LY96, **(B)** sorafenib-PIK3CA, **(C)** sorafenib-NT5E, **(D)** sorafenib-CASP8, **(E)** sorafenib-IFNB1, **(F)** sorafenib-BAX].

The binding energy is an indicator to assess the magnitude of the affinity between the ligand and the receptor. It has been shown that there is strong binding activity between ligand and receptor when the binding energy is less than −5.0 kcal/Mol (He et al., 2023). In this study, the molecular docking results showed that the binding energies between Sorafenib and the target proteins were all less than −5.59 kcal/Mol (Table 2). This suggests that Sorafenib binds strongly to target proteins and that Sorafenib may exert its anticancer effects through six ICD-related genes (Figure 10).

**TABLE 2:** Basic information on the molecular docking between sorafenib and the target protein.

Element	Chemical formula	Molecular weight	Target name	PDB ID	Binding energy(kcal/Mol)
Sorafenib	C_21_H_16_ClF_3_N_4_O_3_	464.825	LY96	2E56	−7.00
			PIK3CA	8EXL	−6.84
			NT5E	7QGO	−6.33
			CASP8	3KJQ	−5.98
			IFNB1	1AU1	−5.61
			BAX	2IMS	−5.59

## Discussions

As a kind of cell death, ICD may have functions in various cancers. To explore the role of ICD in HCC, we retrieved the data from public databases such as TCGA and GEO. We divided the patients into ICD high group and ICD low group, then DEGs and survival analysis were accomplished. We calculated the risk score and constructed a prognostic model which could help predict the prognosis of HCC patients.

From the DEGs, we found that the survival status of HCC patients with HSP90AA1 high expressions was poor. A previous study had found that in HCC patients the HSP90AA1 transcripts in serum were significantly upregulated especially in late HCC patients (Toraih et al., 2019). From the 10 DEGs between HCC and normal liver tissues, we found that ectonucleoside triphosphate diphosphohydrolase-1 (ENTPD1) was higher expressed in HCC compared to normal tissue. ENTPD1 expressed on CD4+Foxp3+ regulatory T cells (Tregs), and in mice, it could inhibit natural killer (NK) cells and promote hepatic metastatic tumor growth, whereas inhibition of the enzymatic activity of ENTPD1 could be used as an adjunct therapy for hepatic malignancies (Sun et al., 2010). C-X-C motif chemokine receptor (CXCR) 3 could induce mobilization and recruitment of Tregs, and it could promote HCC recurrence and enhance acute-phase liver graft injury after liver transplantation (Li et al., 2016). The cytoplasmic level of protein-disulfide isomerase-associated 3 (PDIA3) was increased in HCC tissues, and it could raise dyskerin pseudouridine synthase 1 (DKC1) expression to promote HCC progression and reduce HCC associated recurrence-free survival rates (Ko et al., 2018).

HLA genes could promote the development of HCC and the expression of HLA genes in the ICD high group and ICD low group were different. In nucleotide analogs, naive patients with chronic HBV infection who have an AA genotype of the HLA-DQA1/DRB1 gene are more likely to develop HCC during entecavir treatment (Kozuka et al., 2018). HLA-DQB1-AS1 can interact with ZRANB2 protein to promote cell proliferation and inhibit apoptosis in HCC (Long et al., 2022). In addition, HLA-DQB1 polymorphisms increase the risk of HCC after hepatis C virus eradication (Miki et al., 2020). The risk of developing HCC in chronic hepatis B patients who have the HLA-DRB1*140101 allele is higher (Jin et al., 2012). We found that in ICD high group HLA gene expression such as HLA-DQB1, HLA-DQB1 and other genes were higher than those of the ICD low group.

Then we calculated the immune-checkpoint associated genes in ICD high group and ICD low group. In liver cancer, a T cell immunoreceptor with immunoglobulin and immunoreceptor tyrosine-based inhibitor motif domains (TIGIT) is a marker for T cell exhaustion (Ostroumov et al., 2021). The anti-tumor immune response can be suppressed by TIGIT, which is an inhibitory molecule on CD8^+^ effector memory T cells, and inhibitors of TIGIT combined with anti-PD1 are promising to reduce PD1 inhibitor resistance (Chiu et al., 2020; Ostroumov et al., 2021). From our study, we found that the immune-checkpoint associated genes such as TIGIT in ICD high group were higher than those of ICD low group.

From DEGs, a prognostic model which included 6 genes (BAX, CASP8, IFNB1, LY96, NT5E and PIK3CA) was constructed by using the LASSO Cox regression analysis. A previous study found that in normal human tissues NT5E-2 was expressed at low abundance, but in cirrhosis and HCC it was significantly upregulated (Snider et al., 2014). NT5E-2 codes CD73S protein which has potential significance for cancer, fibrosis and other diseases (Snider et al., 2014). In addition, compared with normal breast tissues, in breast cancer more NT5E gene was methylated, and NT5E gene methylation was associated with breast cancer development and poor prognostic factors, which indicated that NT5E gene methylation may be used as an epigenetic biomarker (Jeong et al., 2020). A previous study from the South Italy population showed that oncogenic mutations were detected in 18 (28%) of the PIK3CA gene from 65 HCC patients, which suggested that at the somatic level mutational activation of the PIK3CA gene can contribute to hepatocellular tumorigenesis (Colombino et al., 2012). In Chinese HCC patients, the frequency of PIK3CA mutations was lower (Li et al., 2012). PIK3CA and Yes-associated protein (Yap) can contribute to liver carcinogenesis by activating the MTORC1/2, ERK/MAPK and Notch pathways (Li H et al., 2015). The risk of PIK3CA gene mutations in patients with chronic Schistosomiasis is higher, which can result in hepatocyte fibrosis and liver cancer eventually (Algabbani, 2022). The polymorphisms of PIK3CA rs17849071 and rs17849079 increase the risk of HCC (Li X et al., 2015). LY96 is significantly upregulated and can be used as a prognostic factor in most types of cancers. LY96 is related to DNA methylation, copy number, microsatellite instability (MSI), somatic mutation, tumor mutation burden (TMB), tumor microenvironment (TME) features and immune cell infiltration in cancers, and LY96 can contribute to drug resistance and regulate classic tumor-associated pathways (Nie et al., 2022). We found the prognostic model constructed by the 6 ICD associated genes could predict the prognosis as well as 1 year, 3 years, and 5 years survival status well. As a tumor-promoting cytokine, IL-17A can regulate alcohol-induced hepatic steatosis, inflammation, fibrosis and HCC, and the development of HCC in alcohol-fed mice was suppressed by deleting the IL-17RA gene (Ma et al., 2020). In HCC patients, increased IL-17 and IL-17RE were related to poorer survival and a higher recurrence rate, and Th17 associated cytokines as well as the crosstalk with various kinds of inflammatory or immune cells might suggest how IL-17-producing CD4+T cells promote the carcinogenesis in HCC (Liao et al., 2013).

We also calculated the immune scores in the two groups. Estimate scores, immune scores and stromal scores in ICD high group were higher than those in ICD low group. Cancer cells, innate and adaptive immune cells, stromal cells, cancer-associated fibroblasts and endothelial cells constitute the TME and elucidate the immune microenvironment can help select appropriate treatment modalities for HCC (Oura et al., 2021). In HCC patients, M0 macrophages can predict overall survival (OS) (You et al., 2022). Compared with normal liver tissues, M0 macrophage infiltrates significantly higher in HCC tissues and it is associated with poor prognosis in HCC patients (Zhang et al., 2022). Higher M0 macrophage infiltrations are related to enriched angiogenesis hallmark genes and decreased OS in patients treated with sorafenib (Farha et al., 2020). We found that macrophage M0 had a positive correlation with the risk scores.

This study had serval advantages. First, we explored the role of ICD in HCC patients which had not been elaborated on previously. Second, the TME, risk scores and ICD were combined to investigate how differences between ICD high group and ICD low group. Third, the prognostic model constructed according to the expression of ICD associated genes could predict the prognosis and survival status in HCC patients. However, there are some limitations as well. Firstly, the prognostic model could not predict the response to immune-checkpoint inhibitors. Secondly, all the data were obtained from a public database, so further studies are still warranted to verify the model in HCC patients.

## Conclusion

In conclusion, in this study we explored the role of ICD associated genes in HCC. The prognosis of HCC patients in the ICD high group was poor than that in ICD low group. A prognostic model of 6 genes including BAX, CASP8, IFNB1, LY96, NT5E and PIK3CA, could predict the survival status and could be used as an independent prognostic factor in HCC patients. This study provided insight into the significance of ICD in HCC, and further studies are needed to validate these findings.

## Data Availability

The original contributions presented in the study are included in the article/Supplementary Material, further inquiries can be directed to the corresponding author.

## Supplementary material

SUPPLEMENTARY FIGURE S1 Correlation analysis of ICD associated DEGs and different immune cells. **(A)** CASP8, **(B)** BAX, **(C)** LY96, **(D)** ENTPD1, **(E)** CXCR3, **(F)** HSP90AA1, **(G)** CALR, **(H)** PDIA3, **(I)** IL6, **(J)** CD4; ICD: Immunogenic cell death, DEGs: Differentially expressed genes).

SUPPLEMENTARY FIGURE S2 Correlation analysis of ICD associated DEGs and related clinical characteristics. (**(A)** age, **(B)** gender, **(C)** N stage, **(D)** M stage, **(E)** BMI, **(F)** AFP, **(G)** T stage, **(H)** pathologic stage, **(I)** vascular invasion, **(J)** race, **(K)** histologic grade, **(L)** fibrosis ishak score, **(M)** residual tumor; ICD: Immunogenic cell death, DEGs: Differentially expressed genes; ns: *p* ≥ 0.05, *: *p* < 0.05, **: *p* < 0.01, ***: *p* < 0.001).
