# Supplementary figures and images for: Comprehensive analysis of immunogenic cell death associated genes expression, tumor microenvironment, and prognosis in hepatocellular carcinoma
Source: Front Pharmacol. 2023 Mar 14;14:1122011. doi: 10.3389/fphar.2023.1122011 (PMC10045985; doi:10.3389/fphar.2023.1122011)

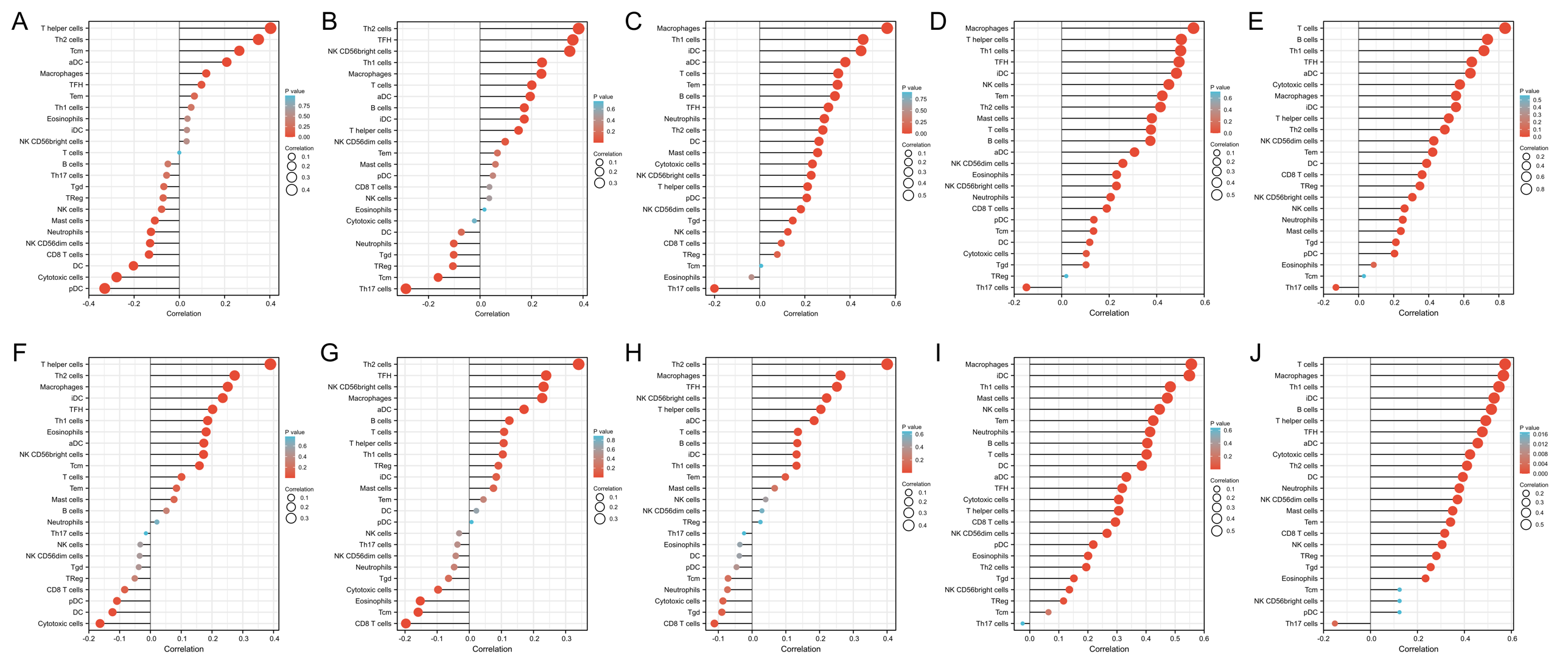

Supplement: Supplementary file 2 [file Image1.TIF]
